# CRISPR/Cas9 and genetic screens in malaria parasites: small genomes, big impact

**DOI:** 10.1042/BST20210281

**Published:** 2022-05-27

**Authors:** Takahiro Ishizaki, Sophia Hernandez, Martina S. Paoletta, Theo Sanderson, Ellen S.C. Bushell

**Affiliations:** 1 Department of Molecular Biology, Umeå University, 901 87 Umeå, Sweden; 2 The Laboratory for Molecular Infection Medicine Sweden (MIMS), Umeå, Sweden; 3 Instituto de Agrobiotecnología y Biología Molecular (IABIMO), INTA - CONICET, Hurlingham, Argentina; 4 Francis Crick Institute, 1 Midland Rd, London NW1 1AT, U.K.

**Keywords:** biochemical techniques and resources, CRISPR, genetics, malaria, *Plasmodium falciparum*

## Abstract

The ∼30 Mb genomes of the *Plasmodium* parasites that cause malaria each encode ∼5000 genes, but the functions of the majority remain unknown. This is due to a paucity of functional annotation from sequence homology, which is compounded by low genetic tractability compared with many model organisms. In recent years technical breakthroughs have made forward and reverse genome-scale screens in *Plasmodium* possible. Furthermore, the adaptation of Clustered Regularly Interspaced Short Palindromic Repeats (CRISPR) and CRISPR-Associated protein 9 (CRISPR/Cas9) technology has dramatically improved gene editing efficiency at the single gene level. Here, we review the arrival of genetic screens in malaria parasites to analyse parasite gene function at a genome-scale and their impact on understanding parasite biology. CRISPR/Cas9 screens, which have revolutionised human and model organism research, have not yet been implemented in malaria parasites due to the need for more complex CRISPR/Cas9 gene targeting vector libraries. We therefore introduce the reader to CRISPR-based screens in the related apicomplexan *Toxoplasma gondii* and discuss how these approaches could be adapted to develop CRISPR/Cas9 based genome-scale genetic screens in malaria parasites. Moreover, since more than half of *Plasmodium* genes are required for normal asexual blood-stage reproduction, and cannot be targeted using knockout methods, we discuss how CRISPR/Cas9 could be used to scale up conditional gene knockdown approaches to systematically assign function to essential genes.

## Introduction

Members of the phylum Apicomplexa are unicellular parasites that cause many important livestock and human diseases including malaria and toxoplasmosis [1, 2]. *Plasmodium* parasites are the causative agents of malaria and infect a wide range of vertebrates, with five species causing disease in humans. There are over 200 million malaria cases annually, resulting in over 600 000 deaths [3]. Recently, the World Health Organization recommended the first malaria vaccine (RTS,S/AS01) for children at risk but its effectiveness is limited and drug resistance threatens the viability of frontline drugs for malaria [3]. There is thus a strong incentive to study gene function in malaria parasites in order to identify new drug and vaccine targets to develop better tools to control and ultimately eliminate malaria.

The *Plasmodium* genomes code for ∼5000 genes, with ∼35% of the protein-coding genes lacking known functional domains revealing their putative function [4]. Knocking out a gene and observing the mutant phenotype is a common and powerful method to understand gene function [5]. Impressive concerted efforts have been made to knockout and phenotype a large number of functionally related genes including 83 *Plasmodium falciparum* putatively exported proteins [6] and 73 predicted *Plasmodium berghei* kinases [7]. However, in order to systematically study gene function at a genome-scale, one has to move beyond the paradigm of knocking out one gene at a time. In 2016, twenty years after the first reported stable transfectant in *P. falciparum*, only 368 of its genes had reported knockout attempts [8], while 529 genes had been targeted in *P. berghei* (analysis of data from RMgmDB: pberghei.eu, [9]). Considerable work has therefore been dedicated to scaling up experiments so that multiple genes can be knocked out in parallel in genetic screens.

The systematic study of gene function in *Plasmodium* has long been hampered by (1) low genetic tractability, (2) the lack of cellular machinery to repair double strand breaks (DSBs) by canonical non-homologous end joining (c-NHEJ) [10], and (3) their highly AT-rich genomes [11]. The lack of c-NHEJ means that all genome manipulation is facilitated by repairing DSBs through homologous recombination, which is an inefficient process. Moreover, it requires the cloning of homology directed repair (HDR) templates of AT-rich DNA that is unstable and difficult to maintain in *Escherichia coli*. During the last decade, several breakthroughs have been made. Methods have been used that either completely circumvent the need for cloning gene targeting vectors [12], or tackle the instability of *Plasmodium* DNA in *E. coli* [13]. Furthermore, the inefficiency of repair by HDR has been at least partly overcome by the impactful adaptation of CRISPR/Cas9 to *Plasmodium* [14] and by the introduction of improved gene targeting vectors [13].

The efficacy paradigm shift brought by CRISPR/Cas9 lies in its ability to precisely engineer a DSB at the target locus, rather than waiting for a break to spontaneously occur. CRISPR/Cas9 gene editing is mediated by the Cas9 endonuclease and the guide RNA (gRNA). The gRNA facilitates site-specific recognition and cleavage of the target sequence by Cas9, generating a DSB that can be repaired by c-NHEJ or HDR [15, 16]. In eukaryotic organisms where the c-NHEJ pathway is intact, DSBs are imprecisely repaired, introducing a mutation at the repair site [17]. Pools of mutants are generated by transfection of complex gRNA pools where c-NHEJ mediated imperfect repair causes parallel loss of gene function. Individual mutants are quantified by sequencing of target specific gRNAs [18]. Guide RNA abundance is taken as a proxy of mutant abundance, where a measurable loss of gene-specific gRNAs is interpreted as an associated fitness cost of inactivating that gene [19]. The circumvention of production of HDR templates together with scalable gRNA synthesis has led to an exponential growth in the deployment of CRISPR/Cas9 screens in c-NHEJ competent organisms [20, 21].

In this article, we review the current landscape of genetic screens in *Plasmodium* and highlight the impact they have had on understanding gene function in malaria parasites. We also summarise the advances that have been brought by the adaptation of CRISPR/Cas9 for manipulation of malaria parasites, and how CRISPR/Cas9 screens have been implemented in the related apicomplexan parasite *Toxoplasma gondii*. Finally, we look ahead to the future challenges and opportunities for CRISPR/Cas9 based genetic screens in malaria parasites.

## Genetic screens in *Plasmodium* parasites

### Genetic screens in *P. falciparum* using chemical and transposon mutagenesis

**Figure 1.**
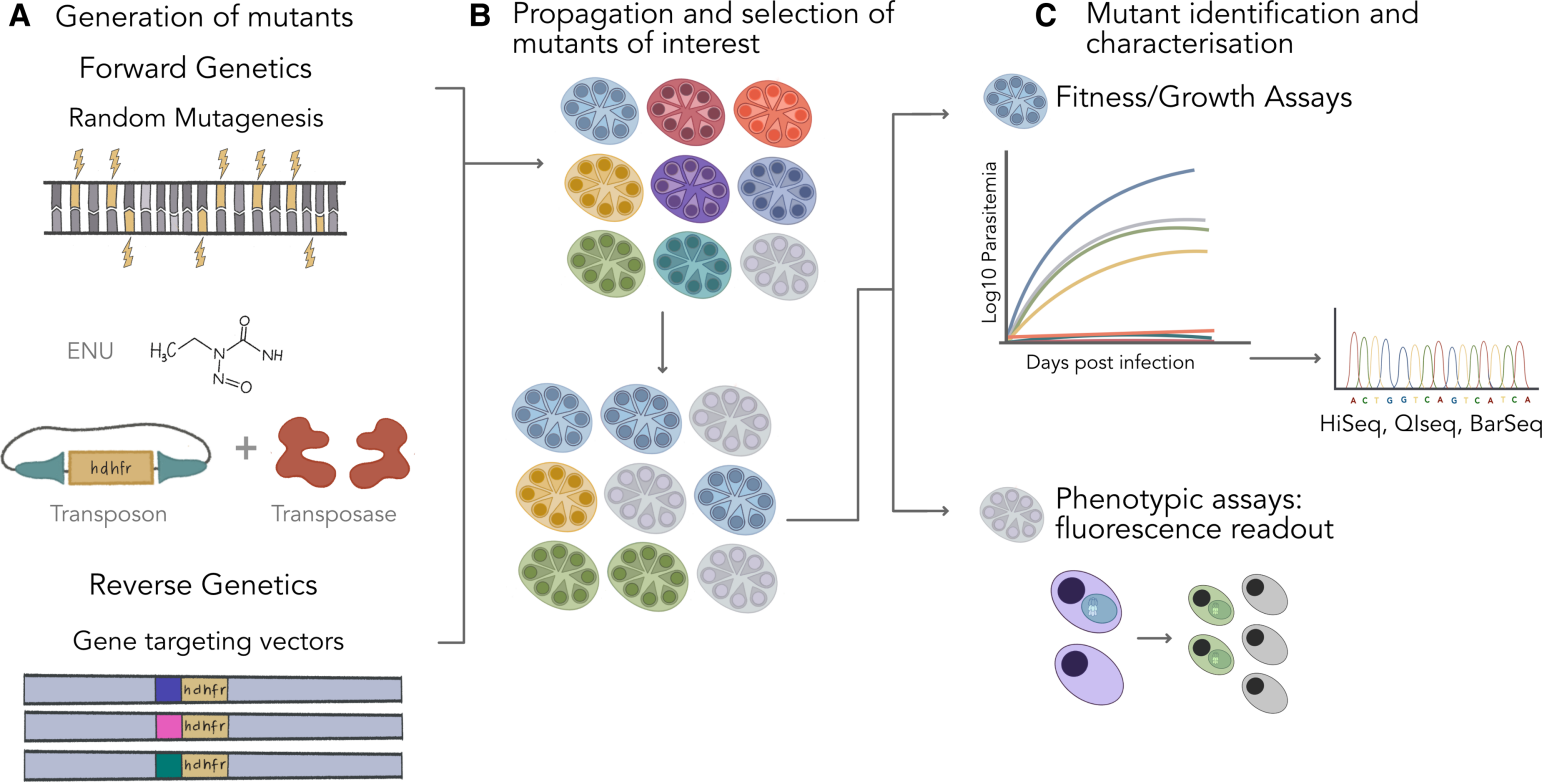
Overview of genetic screens in malaria parasites. (**A**) Pools of mutants have been generated either through random mutagenesis using N-ethyl-N-nitrosourea (ENU), [23] or the *piggyBac* transposon system [24], (forward genetics) or with long homology arm gene targeting vectors [25], (reverse genetics). (**B**) Pools of mutants were selected for and propagated. (**C**) Mutant phenotypes were analysed through growth assays or other phenotypic assays such as microscopy or fluorescence activated cell sorting (FACS), where next-generation sequencing based methods were used to identify and or quantify the mutants. Quantitative insertion sequencing (QIseq), barcode sequencing (BarSeq). Here forward genetics refers to screens where genetic targets are not predetermined and reverse genetics where genetic targets are predetermined by the use of gene targeting vectors.

In forward genetics, the aim is to associate a given phenotype either with natural or randomly introduced genetic variation. These methods were particularly powerful before the arrival of whole genome sequencing and targeted gene disruption. However, forward genetics still plays an important role in scaling up functional genetics (Figure 1). Compared with sequence-directed approaches, it has the advantage of avoiding the challenges of making AT-rich *Plasmodium* gene targeting vectors and the low integration efficiency associated with double homologous crossover. Low integration efficiency is particularly limiting for *P. falciparum* and not entirely overcome by CRISPR/Cas9. Both N-ethyl-N-nitrosourea (ENU) chemical and transposon mutagenesis has been successfully employed to study gene function in *P. falciparum*.

ENU mutagenesis is an effective way to introduce point mutations throughout a genome and has been used to understand the biogenesis of the apicoplast, a vestigial, non-photosynthetic chloroplast present in almost every apicomplexan species. The apicoplast has a 35 kb plastid genome and its maintenance and function require the import of hundreds of nuclear encoded proteins. Nevertheless, in *P. falciparum* the apicoplast loss can be completely compensated by supplementation with isopentenyl pyrophosphate (IPP), [22]. ENU has been elegantly combined with IPP chemical apicoplast rescue and a fluorescent reporter for apicoplast loss to identify 12 novel genes essential for apicoplast biogenesis [23].

An alternative approach is to introduce random genomic alterations using genome-modifying enzymes expressed in the parasite. The lepidopteran *piggyBac* transposon has a TTAA target site that is common in *Plasmodium* genomes. It has been equipped with a human dihydrofolate (*hdhfr*) selectable marker, which upon co-transfection with a transposase expressing plasmid, allows for selection of frequent insertions across the genome [12, 26]. This *piggyBac* system has been successfully combined with quantitative insertion site sequencing (QIseq), [27] to measure the fitness effect of 38 000 mutants representing insertions into 87% of all *P. falciparum* genes during asexual blood-stage growth *in vitro* [24]. At a saturating frequency of insertions across the genome, absence of transposon insertion in any one gene is used as a proxy for gene essentiality. This study represents the first genome-wide genetic screen in *P. falciparum* and identified 2680 genes that are essential for *P. falciparum* asexual blood-stage growth *in vitro* [24]. The *piggyBac* screening system allows for broad analysis of gene function on a genome scale and provides an excellent starting point to study gene function. However, as with any screen, results may contain false-positives and negatives and studies at the single gene level are required to confirm the function of individual genes. In particular, the *piggyBac* system relies on the ability of the transposon to integrate into a particular gene to demonstrate dispensability, which might lead to artifactual calls of essentiality in regions of low chromatin accessibility, and for shorter genes with fewer TTAA target sites.

Layering genetic disruption with other perturbations such as small molecules and raised temperature is a powerful way of revealing growth phenotypes beyond those observable under standard growth conditions [28, 29]. This rationale has been applied to identify the genetic basis of fever tolerance in *P. falciparum* [30, 31]. By subjecting a subset of the *P. falciparum piggyBac* viable mutant library to heat shock conditions that simulate fever, more than 200 heat sensitive mutants were identified. Temperature sensitive mutants were enriched in insertions into known heat shock response genes including those related to protein folding and vesicle trafficking as well as DNA damage and repair [31]. The screen data together with transcriptional analysis uncovered an unexpected link to isoprenoid biosynthesis in the apicoplast, pointing to an algal evolutionary ancestry of the malaria parasite's ability to adapt to febrile temperatures. Furthermore, the same machinery likely facilitates the development of artemisinin resistance [31].

### Genetic screens in *P. berghei* made possible by large scale gene targeting vector libraries

In reverse genetics, the mutation is predetermined by the gene targeting vector (Figure 1). Sequences homologous to the insertion site serve as a repair template and allow the vector to integrate at the target locus by homologous recombination, which is facilitated by the parasite's own molecular machinery for DNA repair [32]. Reverse genetic screens require tackling both low vector integration efficacy in *Plasmodium* and difficulty in cloning AT-rich gene targeting vectors. CRISPR/Cas9 substantially improves the low efficiency associated with homologous recombination. However, vector integration efficiency can also be significantly enhanced by increasing the length of the HDR template [13]. Nonetheless, >2 kb *Plasmodium* DNA fragments are difficult to clone and stably maintain in *E. coli,* and scaling up restriction-ligation cloning is impractical. The challenges of generating long homology arm vectors at scale have been addressed by the use of large-insert (8 kb) *P. berghei* genomic libraries in a linear low-copy vector (pJAZZ, Lucigen). These libraries can be converted into transfection-ready gene targeting vectors by a scalable restriction-ligation free method [13]. The *Plasmodium* genetic modification (*Plasmo*GEM) project has used this method to generate knockout vectors for over 3000 *P. berghei* genes.

*Plasmo*GEM vectors integrate efficiently into the *P. berghei* genome in the absence of episomes (persisting unintegrated vectors) and are equipped with next-generation sequencing readable barcodes. Pools of 100–200 barcoded vectors can be transfected in parallel and results in pools of mutants growing in the bloodstream of a single infected mouse. Mutant barcode abundance is measured along a time course using next-generation sequencing and individual mutant blood-stage fitness can be inferred [33]. This method is termed barcode sequencing (BarSeq) and has been used to map the blood-stage growth phenotypes for over half of all genes in the *P. berghei* genome, revealing a surprisingly high degree of gene essentiality. Sixty-four percent of *P. berghei* genes contribute to normal asexual blood-stage replication *in vivo,* with 45% of genes being essential to this process [25].

Genetic screens provide a priori knowledge of all genes that can be knocked out and thereby be targeted in subsequent phenotypic screens. This rationale was applied in a screen aimed at identifying the genes required for *P. berghei* liver stage development *in vivo.* An analysis of 1342 blood-stage viable mutants [25] identified 461 genes that are needed for optimal transmission of the parasite through the mosquito and onto the subsequent host. Combining BarSeq screening data with metabolic modelling provided a holistic view into the specific metabolic requirement of parasite liver stage development. Seven metabolic pathways were identified as essential for *P. berghei* proliferation in hepatocytes: type II fatty acid synthesis and elongation, and the metabolism of amino sugars, heme, shikimate, tricarboxylic acid, and lipoate [34]. Similarly, a set of 1302 blood-stage *P. berghei* viable mutants were screened for genes important for sexual development. Pools of barcoded mutants were generated in a fluorescent reporter line with green male and red female gametocytes, facilitating the combination of fluorescence-activated cell sorting (FACS) with BarSeq to identify mutants failing to generate male, female or both sex gametocytes. This study pinpointed 30 genes required for the formation of both male and female gametocytes, and 21 genes required exclusively for female and 14 for male gametocytogenesis including regulators expressed very early in sexual development (preprint, [35]).

### Impact of genetic screens in malaria parasites

**Figure 2.**
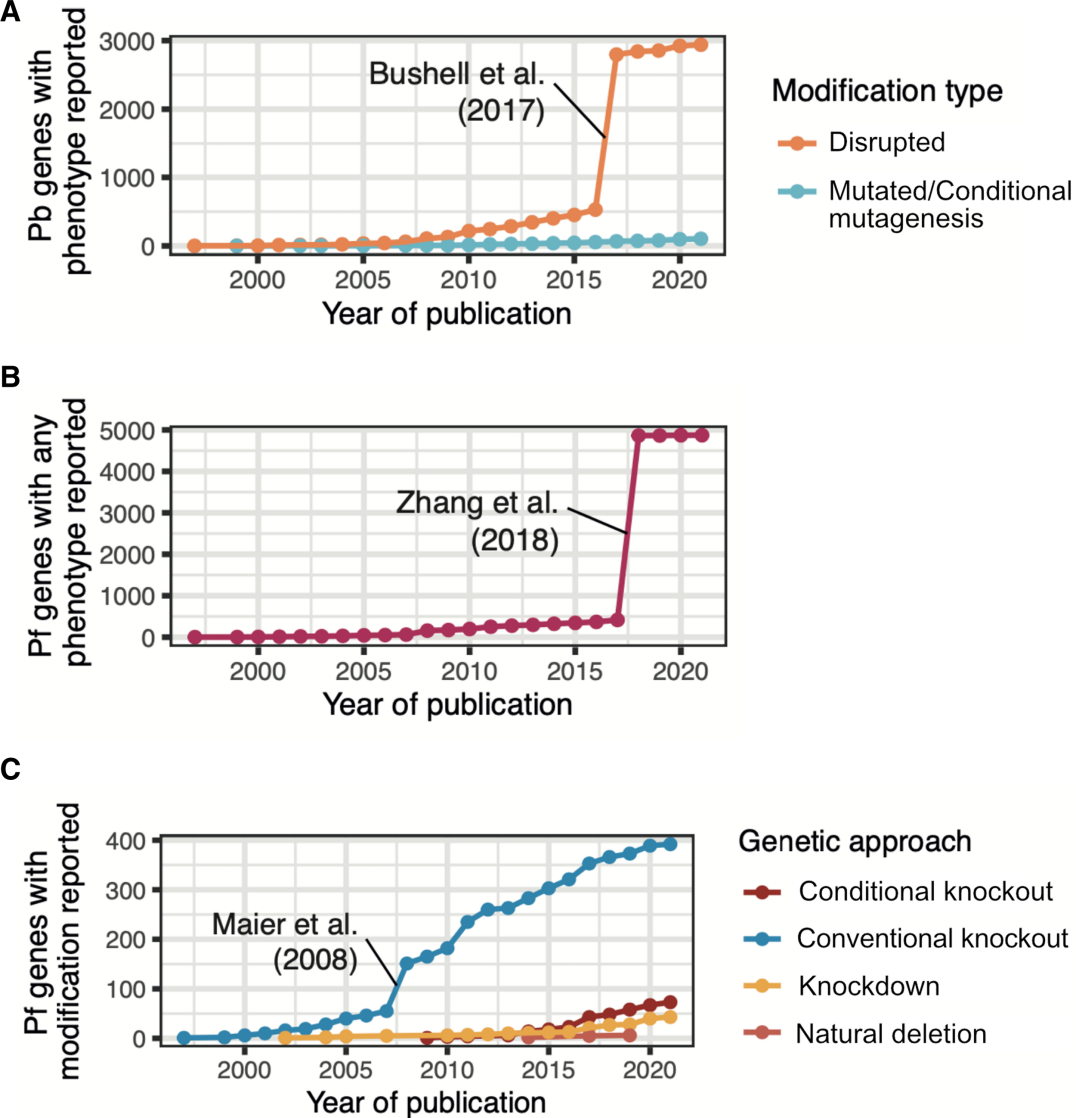
The impact of genetic screens in malaria parasites. Timeline of number of genes with phenotypes reported in (**A**) *P. berghei* (Pb) using data from the rodent malaria genetically modified parasite database [RgMDb, release 2022]. The inflection point from the Bushell et al. (2017), [25] genome scale screen is indicated. (**B**) *P. falciparum* (Pf) using data from PhenoPlasm [Phenoplasm, release 2022]. The inflection point from the Zhang et al. [24] genome scale forward genetic screen is indicated. (**C**) *P. falciparum* split by the genetic approach used, and excluding insertional mutagenesis (i.e. the Zhang et al. screen), showing a recent rise in the use of conditional knockout and knockdown approaches, and with the inflection point from the Maier et al. [6] knockout study indicated. Conditional knockout refers to deleting the gene at a specific time point/stage using dimerisable Cre-recombinase (DiCre). Knockdown here refers to a method where expression is inhibited at either the transcriptional, post-transcriptional, translational level, or the protein is inactivated or mislocalised. Natural deletion refers to spontaneous gene loss in e.g. *in vitro* culture.

The adaptation of screens to *Plasmodium* parasites has had a significant impact on the number of genes that are now associated with a disruption phenotype (Figure 2). Early genetic work at scale targeted sets of genes identified using bioinformatics, such as the parasite's exportome [6] and kinome [7, 36] and made a significant impact on the number of genes for which phenotypes were known. Nevertheless, at the rate of historical phenotype publication under conventional phenotyping approaches it would have taken a good part of a century for genome-wide coverage to have been achieved in *Plasmodium*. The use of large scale screening in *P. berghei* and *P. falciparum*, with the approaches discussed above [24, 25], in a stroke increased the number of phenotypes available by 5-fold and 10-fold, respectively (Figure 2a,b). In both *P. falciparum* and *P. berghei* a high degree of the genome (∼50–60% of all protein coding genes) is required for normal growth, irrespective of screens being conducted *in vitro* or *in vivo*. This has been attributed to the parasite's obligate intracellular lifestyle where it resides within a homeostatic niche and scavenges nutrients from its host, which has resulted in a highly reduced genome [25]. Importantly, the screens showed that many genes lacking functional annotation are important for parasite blood-stage proliferation [24, 25], with 345 conserved hypothetical *P. berghei* genes displaying a blood-stage growth phenotype (PlasmoDB, release 55). Many of these essential genes with unknown function likely represent unique *Plasmodium* biology that can be targeted by drugs with less risk of off-target effects [4]. In contrast with the high degree of essentiality across the genome, 70.2% exported parasite proteins show no observable growth phenotype when deleted *in vivo* [25]. Although these exported proteins likely provide plasticity and support rapid parasite proliferation and immune evasion in diverse hosts, the functional redundancy of many exported proteins accounts for at least some of the difficulty in developing effective vaccines. Understanding the reasons that each essential gene is essential will require a similar step increase in the scale of conditional systems that are now being deployed (Figure 2c).

Tailoring screens to answer specific biological questions, combining screening with additional perturbations, and targeted deep phenotyping readily reveal diverse parasite developmental and adaptive phenotypes. These studies have opened new windows into parasite biology and evolutional ancestry of fundamental processes such as sexual development, adaptation to fever and development of drug resistance. Ultimately, these screens have helped characterise many genes with unknown functions and implicated them in known biological processes for the first time.

## CRISPR/Cas9 in apicomplexan parasites

### CRISPR/Cas9 mediated gene editing in *Plasmodium*

Genetic systems for CRISPR/Cas9 mediated editing of the human malaria parasites *P. falciparum* [14, 37] and *Plasmodium knowlesi* [38] as well as the rodent malaria parasites *P. berghei* [39, 40] and *Plasmodium yoelii* [41–44] are well established. With c-NHEJ pathways absent from *Plasmodium*, CRISPR/Cas9 gene editing requires a third component, the HDR template [32, 45]. The repair template can be cloned into the same circular plasmid that delivers the gRNA [14], or as a separate circular [37] or linear repair template [38–40, 46]. CRISPR/Cas9 combined with HDR is commonly used to knockout genes, or to introduce epitope tags and single point mutations with the desired edit present on the repair template. The implementation of CRISPR/Cas9 to malaria research and detailed consideration of design parameters for efficient genome editing of malaria parasites is comprehensively reviewed elsewhere [45, 47].

### CRISPR/Cas9 screens in *T. gondii*

**Figure 3.**
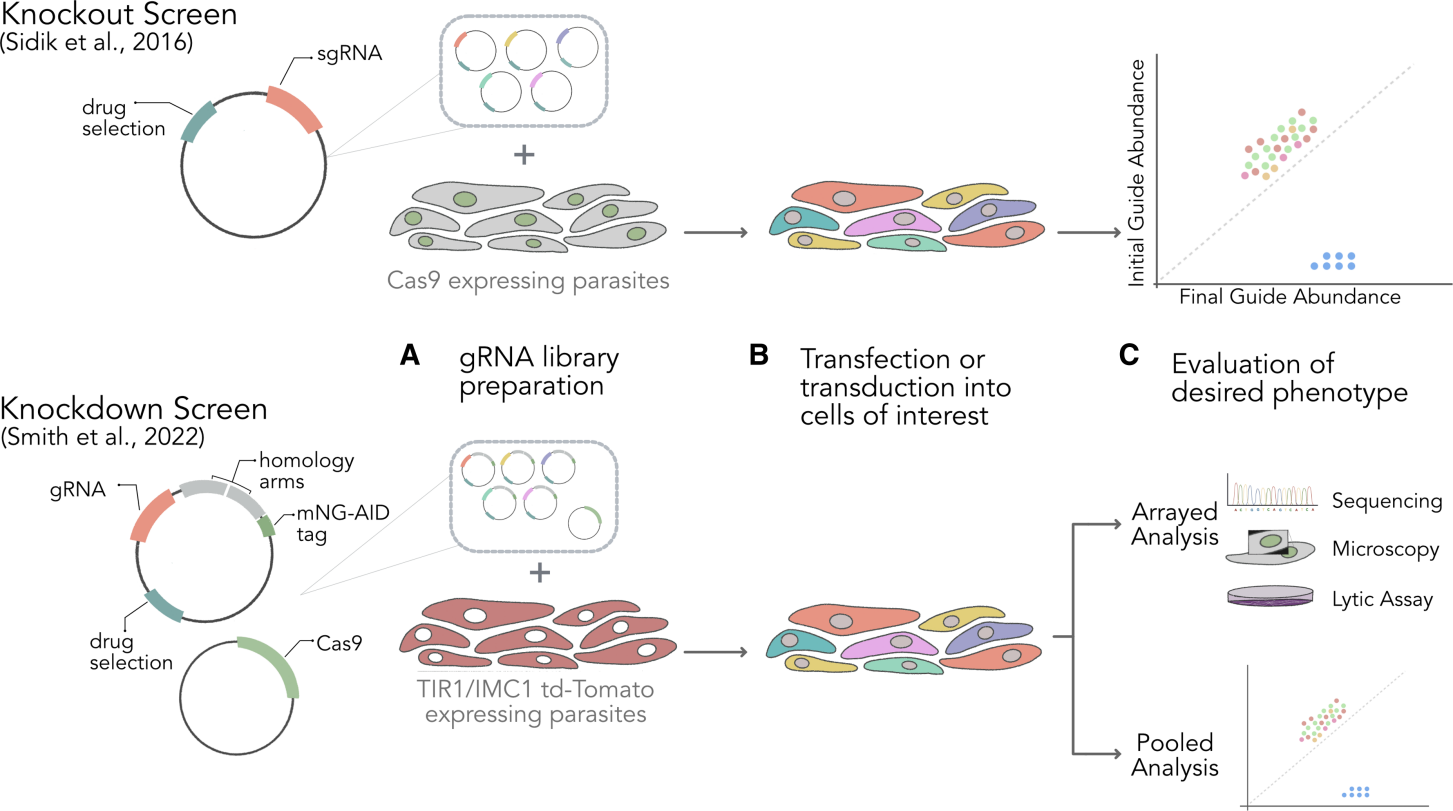
Overview of CRISPR/Cas9 Screens in *Toxoplasma gondii*. Common to all CRISPR/Cas9 screening strategies is the construction of gene specific gRNA vector libraries. Cas9 can be expressed off the same vector as the gRNA or off a separate, co-transfected vector. Alternatively, the Cas9 enzyme can be integrated into the genome. For systems relying on the repair of Cas9-induced DSBs by homologous recombination the HDR template has to be supplied. Top panel: CRISPR–Cas9 knockout screen in *T. gondii* where (**A**) gRNA pools were prepared and (**B**) transfected into Cas9 expressing parasites that produce a decoy gRNA to minimise Cas9 toxicity. (**C**) gRNA sequencing was performed to identify genes important for parasite fitness *in vitro* [49]. Bottom panel: CRISPR/Cas9 knockdown screen in *T. gondii* where CRISPR/Cas9 was combined with the Auxin inducible degron (AID) system. (**A**) Pools of vectors carrying the gRNA and a repair template that introduce a mNeongreen (mNG)-AID tag were co-transfected with a Cas9 expressing plasmid. (**B**) Vector pools were transfected into a TIR1/IMC1 td-tomato line and allowed to propagate before arrayed and (**C**) pooled phenotypic analysis. The AID-tag targets the tagged protein for degradation by exogenous expression of transport inhibitor response 1 protein (TIR1). Inner membrane complex 1 (imc1) td-tomato expression was used in phenotypic assays [55].

In *T. gondii* Cas9 induced DSBs are effectively repaired by c-NHEJ [48], which has paved the way for CRISPR/Cas9 screens in *T. gondii* (Figure 3). The very first genome-wide knockout screen in Apicomplexa was a CRISPR/Cas9 screen mapping all *T. gondii* protein encoding genes required for parasite infection of fibroblasts *in vitro* [49]. The screen identified 200 essential genes specific to, and conserved among Apicomplexa. These genes were termed indispensable conserved apicomplexan proteins (ICAPs) [49]. In contrast with the higher degree of genes that contribute to normal growth of *Plasmodium* (∼50–60%), only 40% of *T. gondii* genes are required for optimal growth *in vitro* [24, 25, 49]. This genome-wide CRISPR/Cas9 screening approach has been extended to identify 22 genes required for growth in the peritoneum and nine genes important for dissemination to organs of *T. gondii* infected mice *in vivo* [50]. Using an alternative approach, an agile system relying on arrayed gRNA libraries that can be combined into smaller gRNA pools to facilitate modular CRISPR/Cas9 screens was deployed to conduct a screen targeted at (exported) effector proteins, which identified several novel genes that are specifically required for intraperitoneal growth in mice [51]. This study also revealed a difference in phenotypes between mutants in a pool and single gene mutant lines, where for a subset of mutants the presence of parasites carrying the wild type allele in the pool compensates and masks the phenotype in a screening format [51]. In addition, CRISPR/Cas9 screens have been combined with additional perturbations such as drug treatment and activation of immune cells to uncover parasite genes underpinning drug resistance [52] and resistance to host defenses [53]. A targeted CRISPR/Cas9 screening of all *T. gondii* metabolic enzymes has also been used together with metabolic modelling to map out *T. gondii* metabolism [54].

## Future perspectives

### Uncovering the function of essential *Plasmodium* genes at scale

Genetic screens have generated a catalogue of essential parasite genes that can potentially be targeted by novel therapeutics. However, it remains challenging to assign molecular function to blood-stage essential genes since no viable knockout mutants can be generated. There are several conditional gene knockout or knockdown tools that can be employed to precisely regulate gene expression or protein activity upon induction. These act at DNA, mRNA or protein levels and those proven to work in *Plasmodium* have been recently reviewed [56]. CRISPR/Cas9 is commonly used to engineer conditional mutants. However, the CRISPR/Cas9 system can also itself be repurposed to act as a transcriptional regulator. The nuclease-dead Cas9 variant (dCas9) lacks endonuclease activity and gRNA mediated binding blocks transcription of the target gene by prohibiting RNA polymerase to proceed. This method is referred to as CRISPR interference (CRISPRi) and has been shown to work in principle in *P. yoelii* [44], *P. falciparum* [57] and *T. gondii* [58].

Attempts have been made to enhance the efficiency of dCas9 by fusion to a histone deacetylase (HDAC) domain to remodel chromatin at the target gene and reduce its expression. Encouragingly, dCas9 fused to *sir2a* resulted in successful gene knockdown in *P. falciparum* [57]. CRISPRi as a system to study *Plasmodium* essential genes at scale is attractive since dCas9 does not induce DSBs and thereby requires only gRNA libraries, which can be used in conjunction with established gRNA sequencing protocols [59]. However, the reported efficiency of CRISPRi is variable [44, 58] and it remains to be seen whether fusion of dCAS9 to an epigenetic regulator is enough to enhance its efficacy to make it applicable at a genome scale. Similar to dCAS9, RNAi can be used to target essential genes that cannot tolerate tagging to introduce conditional alleles and it does not require the production of gene targeting vectors. *Plasmodium* parasites lack the molecular machinery required to execute canonical RNAi [60]. However, a non-canonical RNAi pathway requiring only Argonaute2 (*ago2*) has shown to be effective upon exogenous stable expression of *ago2* in *P. berghei.* Repression of endogenous *P. berghei* genes and recapitulation of their knockout phenotypes were reported [61]. Nevertheless, *ago2* expression was associated with some loss of parasite fitness, and its application across a broader set of genes has yet to be tested [61].

### A path towards CRISPR/Cas9 screens in Plasmodium

The lack of c-NHEJ represents a major hurdle to establishing CRISPR/Cas9 screens in *Plasmodium*. When no homologous repair template is available, *Plasmodium* parasites can use an alternative end-joining mechanism [10, 62]. This mechanism functions at a low frequency but inserts additional nucleotides at the break site, which can generate frameshifts [10] and is likely mediated by microhomology-mediated end joining [62]. A CRISPR/Cas9 strategy based on microhomology-mediated end joining has been utilised to edit the central repeat region of circumsporozoite protein (*csp*) [63]. It remains to be seen if this approach can be made more broadly applicable and potentially exploited for CRISPR/Cas9 gene disruption screens.

Alternatively, to scale up CRISPR/Cas9 reliant on repair by HDR, the gRNA and repair template for each gene must be physically linked. A recent study in *T. gondii* elegantly solves this through a strategy where the gene-specific gRNA and homology arms are held on a single synthetic fragment that can be cloned into the vector in a scalable reaction, generating a final vector that holds an exogenous epitope tag that upon integration modifies the target gene to facilitate its conditional degradation (Figure 3) [55]. Another recent approach to assay the function of essential genes in *T. gondii* uses a screening format that relies on the conditional split-Cas9 system where Cas9 is split into two subunits, each fused to FKBP or FRB domains that, in the presence of rapamycin, come together to restore Cas9 nuclease activity [64]. This strategy allows tight and temporal control of gene editing and permits the study of essential genes in asexual stage mutant phenotypes that would otherwise be lost in a conventional knockout screen using Cas9 pooled format. For example, following synchronisation of conditional *Plasmodium* mutants during asexual blood-stage growth, gene excision can be induced and the timing of mutant arrest during the intraerythrocytic cycle can be assayed.

Malaria control and eradication efforts are under threat from resistance to artemisinin-based combination (ACT) therapies in Southeast Asia, and increasing incidences of emerging artemisinin resistance in Africa [65]. Genetic screens have greatly contributed to our knowledge of *Plasmodium* gene function and shone a light on the function of many parasite genes lacking functional annotation. The screens have identified genes important for proliferation across the parasite life cycle, with the high degree of gene essentiality presenting an abundance of novel therapeutic targets. In addition, combining genetic screens with anti-malaria drug treatment at sublethal doses show great promise for uncovering molecular mechanisms of drug resistance, which might serve to extend the life of existing and future drugs. To assist prioritisation of essential genes for drug development we must better understand their molecular function, while scaling up of conditional approaches remains a challenge. Bridging the existing gaps to implement CRISPR/Cas9 screens to the study of malaria parasites will greatly help to scale up such efforts. CRISPR/Cas9 screens could serve as important tools to fill in gaps left by existing *P. berghei* vector resources and screens, to develop reverse genetic screens for *P. falciparum* and to conduct conditional knockdown screens in both species.

## Perspectives

- The implementation of genetic screens to the study of *Plasmodium* has significantly increased the number of genes associated with a phenotype and revealed a high degree of gene essentiality. These screens shine new light on parasite biology and present a catalogue of new potential drug targets.
- The parallel development of efficient CRISPR/Cas9 gene editing tools for *Plasmodium* and CRISPR/Cas9 screens in the related apicomplexan *Toxoplasma gondii* forms a foundation for developing CRISPR/Cas9 screens in *Plasmodium*, which to date has not been feasible.
- *Plasmodium* CRISPR/Cas9 screens must overcome the lack of non-homologous end joining. Enhancing the efficacy of existing micro-homology mediated end joining or physically linking repair template and gRNA in a single scalable vector could offer a way forward. Efforts towards developing conditional methods to understand the function of essential genes at scale would be of special merit.

## Abbreviations

DSBs: double strand breaks
ENU: N-ethyl-N-nitrosourea
FACS: fluorescence-activated cell sorting
HDR: homology directed repair
IPP: isopentenyl pyrophosphate
